# Reorganization of Burst Initiation Zones as a Signature of Stimulation-Induced Plasticity in In Vitro Cortical Networks

**DOI:** 10.1523/ENEURO.0457-25.2026

**Published:** 2026-07-31

**Authors:** Mikel Ocio-Moliner, Anna-Christina Haeb, Akke Mats Houben, Daniel Tornero, Jordi Soriano

**Affiliations:** ^1^Departament de Física de la Matèria Condensada, University of Barcelona, Barcelona 08028, Spain; ^2^University of Barcelona Institute of Complex Systems (UBICS), Barcelona 08028, Spain; ^3^Laboratory of Neural Stem Cells and Brain Damage, Department of Biomedical Sciences, Institute of Neurosciences, University of Barcelona, Barcelona 08036, Spain; ^4^Institut d’Investigacions Biomèdiques August Pi i Sunyer (IDIBAPS), Barcelona 08036, Spain; ^5^Centro de Investigación Biomédica en Red Sobre Enfermedades Neurodegenerativas (CIBERNED), Madrid 28029, Spain

**Keywords:** effective connectivity, in vitro, microelectrode arrays, network bursting, neuronal culture, plasticity

## Abstract

The spontaneous activity of neuronal cultures is dominated by periods of coherent activity known as *network bursts*. The dynamics of the initiation and propagation of these bursts are believed to depend on the structural connectivity of the neuronal network. Specifically, the spatial loci of burst initiation, referred to as initiation zones (IZs), are shaped by a complex interplay between dynamics and connectivity, with the particular structure of the latter playing a crucial role. Thus, alterations in network structure, such as those following from plasticity mechanisms activated by perturbations, are expected to lead to changes in network burst dynamics. To shed light on this point, we investigated the effect of localized electrical stimulation on the initiation of network bursts in in vitro cortical networks from embryonic rats of either sex cultured on high-density microelectrode arrays, and observed that electrical stimulation significantly altered the spatial patterning of IZs. To explore the mechanisms underlying these alterations, we assessed changes in effective connectivity (EC) using transfer entropy. Although both IZs and EC exhibited similar remodeling following stimulation, EC alone could not fully account for the observed IZ shifts. Moreover, IZ remodeling was largely unpredictable and independent of the stimulation site, suggesting that the observed changes were due to large-scale remodeling of connectivity structure, likely involving local potentiation/depotentiation and homeostatic mechanisms. Our findings provide evidence that electrical stimulation can remodel burst initiation and effective connectivity, although achieving precise local control appears unattainable.

## Significance Statement

Spontaneous bursts in neuronal cultures emerge from a complex interplay between connectivity and intrinsic neuronal dynamics. Here, we show that brief electrical stimulation can modulate this interplay by shifting the spatial zones where bursts originate. Using high-density microelectrode arrays, we find that burst initiation zones are highly sensitive to stimulation, exhibiting pronounced and lasting spatial changes even when global firing rates remain stable. Effective connectivity maps reveal a coordinated but subtle network remodeling, suggesting that widespread synaptic adjustments and homeostatic mechanisms underlie these changes. Our results highlight both the responsiveness of spontaneous network activity to perturbations and the difficulty of achieving precise spatial control using simple stimulation protocols.

## Introduction

Biological neuronal networks form synaptic connections that evolve into self-organized, functional circuits capable of both electrical and chemical signaling. A hallmark of developing cortical networks is the presence of spontaneously synchronized bursting events, where large neuronal populations fire in coordinated waves (Wu et al., 2024). Bursts are a conserved phenomenon across neuronal systems, from dissociated single-cell cultures and acute slices, to large-scale in vivo structures such as the thalamus, cortex, and hippocampus, and are observed across species from invertebrates to mammals (Khazipov and Luhmann, 2006; Luhmann et al., 2016).

Network bursts play an important role in the maturation of neuronal networks in vivo. During development, highly synchronized activity patterns emerge (Dupont et al., 2006; Blankenship and Feller, 2010; Lakhera et al., 2025) that promote connectivity and circuit refinement. Postnatal sensory stimuli refine structural connectivity and suppress widespread bursting, driving circuits toward functional specialization. In contrast, dissociated in vitro networks lack sensory input and display a markedly different behavior of “arrested development,” characterized by persistent network-wide bursting that emerges as soon as neurons and synapses form. These network bursts persist as a dominant activity pattern throughout the culture’s lifespan, although they change in timing and spatiotemporal complexity (Khazipov and Luhmann, 2006; Blankenship and Feller, 2010; Luhmann et al., 2016) with network growth (Wagenaar et al., 2006a) and evolving excitatory–inhibitory balance (Voigt et al., 2001; Crocco et al., 2025).

The predominance of network bursts in vitro—and their importance during development in vivo— has driven research aimed at understanding the mechanisms underlying their formation and regulation. This topic has gained particular relevance in recent years with the rise of biological neurocomputation, where spontaneous network-wide bursts can strongly influence input–output relationships (Houben et al., 2026) and the induction of plasticity during training. A key step in understanding burst formation is to identify the regions where bursts originate. Crucially, bursts do not activate the entire network simultaneously, but rather emerge from specific sites within the culture and rapidly propagate through connected neurons. These sites —referred to as *burst initiation zones (IZs*; Eytan and Marom, 2006)—correspond to the areas where activity nucleation is highly localized, both in time and space.

The phenomenon of burst initiation can be understood within the *noise focusing* framework (Orlandi et al., 2013). In this view, intrinsic biological noise interacts with the network’s topological structure. Weak, random inputs are amplified by network motifs, local groups of interconnected neurons, that lower the threshold for neuronal firing and steer activity toward the IZs. Consequently, spontaneous synaptic noise can be locally amplified by recurrent connectivity, gradually recruiting larger neuronal ensembles until a coordinated network burst emerges. A complementary perspective on the organization of burst initiation is provided by the boxing model (Feinerman et al., 2007), which captures the competition between multiple IZs, predicting two dynamical regimes: one where a single zone dominates over its competitors, and another where several IZs coexist.

Within these frameworks, burst initiation strongly depends on network connectivity. For instance, the distribution of burst IZs changes along development (Montalà-Flaquer et al., 2022) and is completely remodeled upon physical damage (Faci-Lázaro et al., 2019; Teller et al., 2020). Thus, an interesting open question that arises is whether milder forms of network remodeling, such as synaptic plasticity, are sufficiently strong to alter burst initiation.

Many studies investigate inducible plasticity using chemical, optical, or electrical stimulation protocols, evaluating changes through parameters of individual recording channels (Chiappalone et al., 2008; Churchland et al., 2010). The advent of high-density multielectrode arrays (HD-MEAs) brought electrical stimulation to the forefront for its capacity to locally induce plasticity while simultaneously assessing network-wide effects. However, the mechanisms by which electrical stimulation produces lasting network-level changes are still not completely understood (Wagenaar et al., 2006b; Massobrio et al., 2015).

In this study, we show that electrical stimulation can induce shifts in IZs of spontaneous network activity, reflecting a remodeling of network connectivity. To this end, we employed dissociated in vitro cortical networks cultured on HD-MEAs, which allow the mapping of electrophysiological signals with high spatiotemporal resolution and provide a powerful platform to study spontaneous neuronal dynamics. Our work provides a formalism for capturing network remodeling via IZs analysis coupled with effective connectivity measures, potentially helping to understand connectivity changes due to stimulation in in vitro neuronal networks (Obien et al., 2015; Parodi et al., 2023; Yang et al., 2023).

## Materials and Methods

### HD-MEA chips preparation

All experiments were carried out using CMOS-based HD-MEA chips (CorePlate 1W 38/60, 3Brain AG), which enable the simultaneous acquisition of neuronal activity in 4,096 electrodes arranged in a 64 × 64 grid. The chip’s electronics is placed at the bottom of 25 mm diameter wells that provide humidity and nutrients to the neurons growing on the chip. All HD-MEA chips were cleaned, handled and protein-coated following the protocols provided by the manufacturer. Specifically, the chip wells were first loaded with 2 ml dH_2_O for 2 h to rehydrate the surface. Next, the wells were sterilized by filling them to the edges with 70% ethanol for 1 h. After rinsing the wells with sterile dH_2_O, the HD-MEA chips were exposed to UV light for 15 min and incubated overnight in cell medium to enhance surface hydrophilicity. To promote cell adhesion, the chip surface was treated with adhesive proteins: first with polyornithine in water (100 μg/ml, Sigma-Aldrich) at room temperature overnight, followed by mouse laminin (5 μg/ml, Merck) at 37°C overnight.

### Rat cortical cultures

All procedures were approved by the Animal Experimentation Ethics Committee (CEEA) of the Universitat de Barcelona (ethical order B-RP-094/15-7125 of July 10, 2015), in accordance with the regulations of the Generalitat de Catalunya (Catalonia, Spain). Cortical neuronal cultures were obtained by mechanical dissection from embryonic day 18 (E18) Sprague-Dawley rat embryos of either sex. Embryonic brain dissections were carried out in ice-cold L-15 medium (Gibco, L1518), enriched with 0.6% glucose and 0.5% gentamicin (Sigma-Aldrich, G1272). After isolating the cortices from the meninges, they were mechanically dissociated through repeated pipetting of the tissue in cell medium, consisting of Neurobasal (Thermo Fisher Scientific, 21103049) with the following supplements: B27 Plus (2%, Thermo Fisher Scientific, A3582801), Fetal Bovine Serum (5%, Thermo Fisher Scientific, 10270106), stable L-Glutamine (1% GlutaMax 100x, Thermo Fisher Scientific, 35050038), and Antibiotic-Antimycotic (0.1%, Thermo Fisher Scientific, A5955). 100 μl of cell suspension was plated on the precoated chip surface (36 mm^2^) at a 2,000 cells/mm^2^ concentration. After 2 h to allow for cell stabilization and attachment to the surface, 1.5 ml of cell medium was added. Cultures were maintained at 37°C, 5% CO_2_, and 95% humidity. Half of the medium was replaced every other day. Spontaneous neuronal activity on the HD-MEA chips emerged by day in vitro (DIV) 6, first in the form of sporadic activity events that rapidly evolved toward collective bursting events.

### Electrical activity recording

Electrical activity on the 3Brain’s HD-MEA chips was recorded on the 4,096 electrodes using the BioCAM DupleX 2019 system (3Brain AG) at a sampling rate of 20 kHz. Each electrode measures 21 × 21 μm^2^ with a 60 μm inter-electrode pitch. Electrodes are arranged in a 64 × 64 grid covering a 3.8 × 3.8 mm^2^ area. The array is embedded in a larger 6 × 6 mm^2^ surface where neurons are also plated and grow, potentially generating activity outside the recording boundaries. Data acquisition and pre-processing were carried out using the BrainWave5 software (3Brain AG), with subsequent offline analysis in MATLAB (MathWorks), see Section “Data analysis.”

### Electrical stimulation protocol

**Figure 1.**
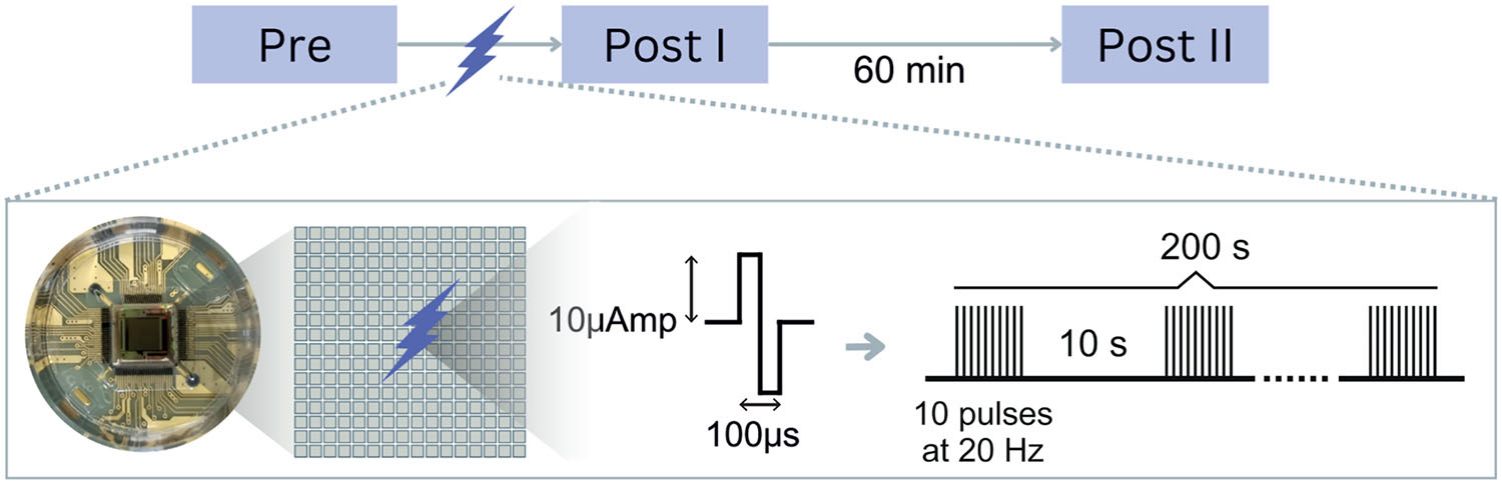
Experimental protocol. Spontaneous activity (“Pre,” “Post I,” and “Post II”) was recorded for 5 min each (blue boxes) on HD-MEAs containing 64 × 64 electrodes. Tetanic stimulation across a preset electrode (blue flash) was performed between the “Pre” and “Post I” recordings, and consisted of a train of 10 pulses at 20 Hz, repeated every 10 s for a total of 200 s. Across the three phases, the number of active electrodes did not significantly vary (Fig. 1-1). In addition, no differences were observed between the three phases when comparing the firing rate, the interneuronal correlation and the Fano factor distributions (Fig. 1-2). Finally, spike sorting was performed to ensure that units’ distributions remained stable across phases (Fig. 1-3).

Electrical stimulation (Fig. 1) was applied as a current delivered to a set of electrodes. Typically, four to five electrodes were used, although larger sets were also tested (see Extended Data). Stimulation was applied only to electrodes meeting two essential criteria: first, they exhibited activity characterized by extracellular spike waveforms; and, second, they participated in at least 90% of the detected network bursts during an exploratory recording prior to the “Pre” phase, which was used to select stable and representative electrodes. From these candidate electrodes a subset was chosen to be stimulated. The criteria to segment bursting activity is detailed in “Global network activity and detection of bursting events.” This selection strategy ensured functional connectivity in the recorded network before any stimulation was applied. The stimulated electrodes were located in the vicinity of one another, typically 2 to 8 electrodes apart. Basic stimulation consisted of bipolar pulses (positive first) of ±10 μA that lasted 100 μs. Trains of tetanic stimulation were then set as bursts of 10 pulses at 20 Hz, repeated every 10 s for 20 times (Fig. 1). This protocol was previously used to induce long-term potentiation (LTP; Jimbo et al., 1999). The stimulus trains were delivered irrespective of the ongoing network activity, thereby uniformly sampling all possible network states (bursting or not) without bias for any particular state.

10.1523/ENEURO.0457-25.2026.f1-1 Figure 1-1 Stability of network activity and dynamics following stimulation. Box plot of the differences in the number of active electrodes (left) and IBIs (right) between Pre and Post II phases for control and stimulated cultures. No significant differences are observed between conditions for both the number of electrodes (*U* = 193, *p* = 0.61, *N_Ctrl_* = 13, *N_stim_* = 33, Mann–Whitney U test) and the IBI (*U* = 190, *p* = 0.44, *N_Ctrl_* = 13, *N_stim_* = 33, Mann–Whitney U test). The variation of active electrodes is not significantly different from zero (Control: *t* = −122, *p* = 1.00, *N_Ctrl_* = 13; *S_tim_*: *t* = −155, *p* = 0.27, *N_Stim_* = 33, Student’s *t*-test)), indicating that there was no cell damage caused by stimulation. For the IBI difference, both distributions are also centered around zero, indicating that stimulation does not substantially alter the intrinsic dynamics of the neuronal cultures (Control: *t* = −0.59, *p* = 0.72, *N_Ctrl_* = 13; Stim: *t* = −0.45 *p* = 0.86, *N_Stim_* = 33, Student’s *t*-test)). The representative experiment from Fig. 6A is highlighted in a darker tone. Download Figure 1-1, TIF file.

**Figure 2.**
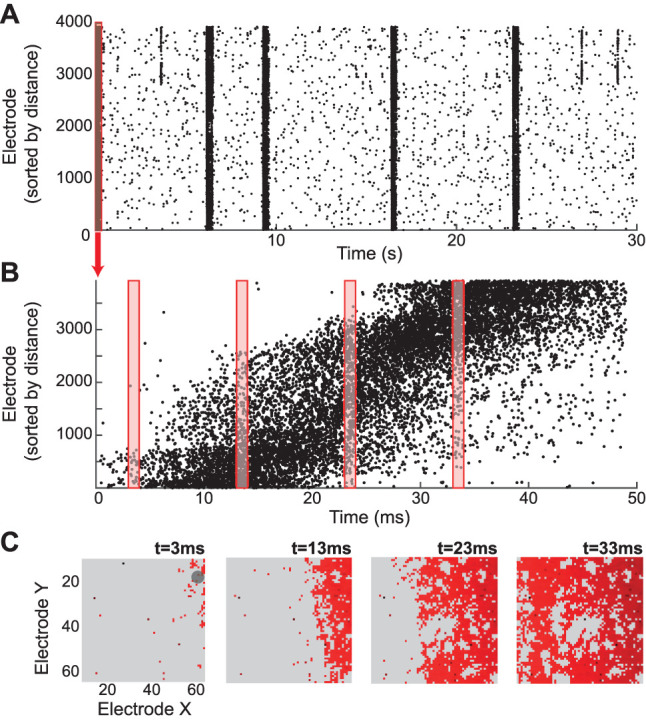
Neuronal network dynamics and bursting. ***A***, Raster plot of spontaneous neuronal activity on the HD-MEA, with each dot representing an individual spike in an electrode. Two different regimes can be observed, one characterized by sparse, random activity, and another one characterized by highly correlated network-wide activity that shapes a network burst (apparent vertical lines). ***B***, Detailed raster plot of the first recorded burst (50 ms window). Electrodes are ordered by their distance from the initiation zone of the collective bursting event, revealing a wave-like propagating front of activity. ***C***, Snapshots of neuronal activity, showing the active electrodes as red squares. Data is binned within a 1 ms window, corresponding to the red boxes of panel ***B***, and is spaced every 10 ms. Electrodes appear in bright red when they spike, and the color progressively fades to darker tones with time to help track the propagation of activity. The burst initiation zone marked with the circle corresponds to the origin of the propagating wave.

10.1523/ENEURO.0457-25.2026.f1-2 Figure 1-2 Stability of network activity statistics across experimental phases. Firing rate (top), Pearson correlation (middle), and Fano factor (bottom) distributions for the ‘Pre’ (left), ‘Post I’ (middle), and ‘Post II’ (right) phases. Metrics were computed separately for activity occurring within network bursts (blue) and outside bursts (red). Across all measures, no qualitative differences are observed between phases, indicating that the overall statistical properties of network activity remain comparable throughout the experiment. Download Figure 1-2, TIF file.

**Figure 3.**
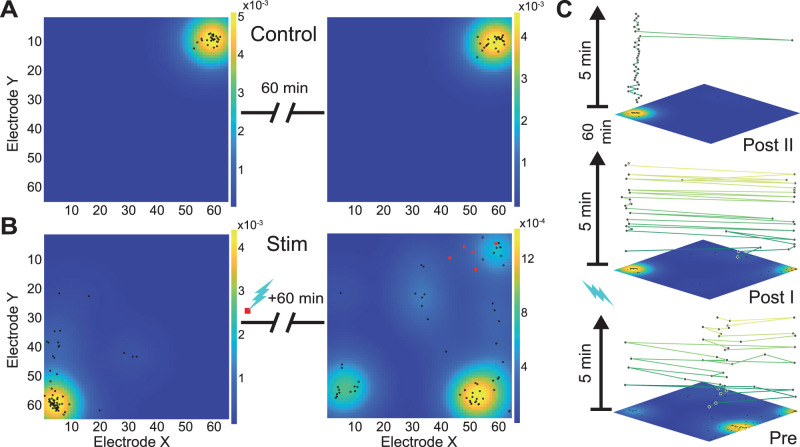
Quantification and evolution of burst IZs. ***A***, Two-dimensional probability density function of IZs for the “Pre” (left) and “Post II” (right) phases under control condition (two consecutive recordings separated by 1 h). Black dots represent the initiation point of each burst through the 5 min recording of spontaneous activity, showing a consistent concentration in the top-right corner with no relevant spatial reorganization over the time span between the two phases. ***B***, Corresponding results for stimulated condition, in which the “Post II” recording is carried out 1 h after delivering the electrical stimulation on preset electrodes (red squares). Here, the family of burst initiation points is more spread and the strongest initiation probability shifts from the bottom-left to the bottom-right corner of the culture. ***C***, Sequence of burst initiation (black dots connected with lines) within the 5 min duration of each recording, for the “Pre,” “Post I,” and “Post II” phases. In the “Pre” phase, burst initiation competes (“boxing model”) between two equally probable hot spots. A different initiation zone emerges at the other edge of the culture during the “Post I” phase, but still competes with a previous one. The new initiation zone finally becomes the dominant one during the “Post II” recording, after a gradual shift.

10.1523/ENEURO.0457-25.2026.f1-3 Figure 1-3 Assessment of homogeneity in neuronal sampling across electrodes. Spike sorting was performed using a standard waveform-based pipeline. Spike waveforms were extracted using a window of 0.5 ms pre-peak and 1.0 ms post-peak. Features were obtained via principal component analysis (first three components), and clustering was performed using k-means with the number of clusters selected using the gap statistic (up to 10 clusters, at least 2 spikes per cluster). **(A)** Distribution of Gini coefficients quantifying the inequality in the number of detected units per electrode. Values were compared against a Monte Carlo null model in which a fixed number of units M were randomly assigned to K electrodes. The observed values for the Gini coefficient (0.27 ± 0.08) did not significantly differ from the null expectation (*T* = 842, *p* = 10−14, *z* = −7.7, *N* = 126, Wilcoxon signed-rank test), indicating no systematic deviation from homogeneous sampling at the network level. **(B)** Distribution of coefficients of variation (CV) of the number of units per electrode. The average CV was 0.55 ± 0.20, which was significantly lower than the value obtained from the multinomial permutation *T* = 1655, (*p* = 10^−8^, *z* = −5.7, *N* = 126, Wilcoxon signed-rank test). **(C)** Relative variation in the number of detected units per electrode (defined as the percentage of the difference over the mean value across the three phases) across experimental phases (Pre–Post I, Post I–Post II, and Pre–Post II). No significant differences were observed (ΔUnits*_Pre−PostI_*=(2 ± 9)%, *t* = −0.7, *p* = 0.13; ΔUnits*_PostI−PostII_*=(0.002 ± 0.023)%, *t* = −0.01, *p* = 0.62; ΔUnits*_Pre−PostII_*=(2 ± 9)%, *p* = 0.17, *t* = −0.9; *N* = 38, Student’s *t*-test). In addition, the CV of the number of units per electrode was 0.02 ± 0.05. Altogether, these results indicate that variability in the number of neurons contributing to each electrode is limited. We can observe how the larger variation occurs right after the stimulation, and it is very stable during the following hour, although none of the changes are significant. Download Figure 1-3, TIF file.

For each recording session on a given neuronal culture, the experimental protocol consisted of the following phases:

1. Pre: 5-min recording of spontaneous activity.
2. Tetanic stimulation for 200 s.
3. Post I: 5-min recording of spontaneous activity.
4. Rest for 60 min.
5. Post II: 5-min recording of spontaneous activity.

In the stimulated and control conditions cultures displayed qualitatively similar dynamics, quantified through the average firing rate (FR) ≃ 2.5 Hz and the inter-burst interval (IBI) ≃ 5 s. No differences were found between the “Pre” and “Post II” phases, neither in the FR nor in the IBI (Fig. 1-1). In addition, the number of active electrodes did not significantly vary between the different phases, indicating that there was no cell damage caused by stimulation (Fig. 1-1). Burst sizes generally involved 80% of the recording electrodes throughout the culture.

In addition, no differences were observed between the three phases when comparing the FR, the interneuronal correlation and the Fano factor distributions during and outside network bursts (Fig. 1-2).

### Dataset and recordings

Table 1 provides a summary of the conducted experiments, as well as the experimental repetitions, for different culture days in vitro. Experiments were typically conducted at DIV 17–24, when the neuronal network was considered mature, exhibiting spontaneous network bursting activity and expressing all major receptor types relevant for plasticity studies, including AMPA, NMDA, and GABA_A_ receptors (Luhmann et al., 2016; Edwards et al., 2017). Further details on the location of the stimulated electrodes are provided in the accompanying Extended Data Table 1-1.

10.1523/ENEURO.0457-25.2026.t1-1 Table 1-1 Extended dataset summarizing stimulation configurations, initiation zone (IZ) estimates, and burst statistics for all recordings. Download Table 1-1, CSV file.

**Table 1.** Number of repetitions for each of the conditions of different recordings

Recording day in vitro (DIV)	Control trials	Stimulated trials
17	3	5
18	1	4
19	5	7
20	2	8
21	1	4
24	1	4
26	–	1
Total	*N*_C_ = 13	*N*_S_ = 13

Further details on the location of the stimulated electrodes are provided in Table 1-1 (Extended Data).

### Data analysis

The data recorded with HD-MEAs was analyzed to identify neuronal activity events (spikes), characterize the spatiotemporal dynamics of bursting events, and compute burst initiation statistics and effective connectivity (EC).

#### Spike detection

Neuronal activations (spikes) from the spontaneous activity recordings were automatically extracted through the BrainWave5 software by applying first a 800 Hz high-pass filter on the raw data followed by a “precise timing spike detection” analysis (Maccione et al., 2009) using the parameters provided in Table 2.

**Table 2.** Parameters for spike extraction using precise timing spike detection

Parameter	Value
Standard deviation factor	8
Peak lifetime period (ms)	2
Refractory period (ms)	2
Spike assignment	Automatic
AI-validation	Off
Pre-peak wave duration (ms)	1
Post-peak wave duration (ms)	1.5
Discard noisy electrodes	On
Discard chip calibration artifacts	On

To assess potential heterogeneity in neuronal sampling across electrodes, we performed spike sorting and quantified the distribution of detected units per electrode. The Gini coefficient and coefficient of variation of units’ distribution were compared against a Monte Carlo null model in which the same number of units were randomly assigned to the 4,096 electrodes. No significant deviation from the null expectation was observed, and units’ distributions remained stable across experimental phases (Fig. 1-3). These results indicate that variability in neuronal contributions does not significantly bias electrode-level analyses.

#### Global network activity and detection of bursting events

Spontaneous activity data was binned at a resolution of 10 ms to smooth out fluctuations. For each time bin, the fraction of active electrodes was then computed to obtain the global activity *A* of the network as a function of time. Peaks in global activity denote episodes of strong network-wide events. To detect these episodes, the mean *μ*_G_ and standard deviation *σ*_G_ of the global activity were computed. A peak was classified as a burst and retained for further analysis when its amplitude exceeded a minimum value of $A_{\min}=\mu_{G}+2.5\sigma_{G}$. The burst was considered to end once the global activity dropped below the background noise level *A*_bgnd_ = *μ*_G_ + 0.5*σ*_G_.

#### Detection of burst IZs

The spatial origins of bursts in the cultures, hereafter referred to as *IZs*, were determined manually using the following procedure. Burst times were detected as described in the previous section, and the activity from the start of the burst until its end was extracted using a custom-made software. Each detected burst was then visualized as a spatiotemporal sequence of spikes that could be played at millisecond resolution. To facilitate tracking the propagation of activity, electrodes were displayed in red when they spiked, with the color progressively fading to a darker tone with time (as in Fig. 2*C*). The initiation point was defined as the location from where the activity initiated and started to propagate. Once identified, this point was manually marked by clicking on the corresponding region of the electrode array. Manual IZ detection was carried out for half of the identified bursts per recording, randomly selected from the full set of identified bursts, and presented in a random order to avoid any serial biases in the IZ identification. Given that a 5 min recording typically contained ∼60 bursts, analyzing half of them provided sufficient sampling of the spatial distribution of initiation points while keeping the manual inspection manageable. The number of analyzed bursts per experiment is shown in the Extended Data table.

This procedure is robust to the spatial subsampling caused by the blind zone detailed in Section “Electrical activity recording”: when bursts originate outside the recorded area, the initiation point is defined as the first location at which activity enters the recording region. While this may introduce a bias toward detecting initiation points near the array boundaries, our analysis focuses on relative changes in IZ location across phases rather than their absolute spatial positions, thereby minimizing the impact of this effect on our conclusions.

Only events exhibiting wave-like propagation patterns (i.e., with an identifiable propagation front) were retained for IZ identification (Fig. 2). These events represented ∼99.8% of all detected bursts. The few excluded cases corresponded to events in which activity percolated quasi-instantaneously across the whole array, preventing the identification of a clear spatial origin. In such cases, the concept of an IZ cannot be defined, and these events were therefore excluded from the IZ analysis.

Other approaches for computing the origin of the bursts, such as Center of Activity Trajectories (Gandolfo et al., 2010), were discarded due to the presence of quasi-simultaneous activations in competing foci, yielding poorly informative trajectories.

Following Orlandi et al. (2013), the set of *N* determined initiation points $\{(x_{0},y_{0}){\}}_{N}$ was converted into a probability density function (PDF) as:

$$P_{\text{IZ}}(x,y)=\sum_{x_{0},y_{0}}\frac{1}{2\pi N\sigma^{2}}\exp\;\left(-\frac{(x-x_{0}{)}^{2}+(y-y_{0}{)}^{2}}{2\sigma^{2}}\right),$$

where the variance *σ* was set to 10% of the recording area. *P*_IZ_ is normalized such that it ranges between 0 and 1.

The maxima of *P*_IZ_(*x*, *y*), i.e., where a high number of initiation points concentrate, will be referred to as the IZs. Multiple maxima of *P*_IZ_(*x*, *y*) can coexist.

#### FR spatial distribution

To characterize the spatial distribution of neuronal activity, a discrete Gaussian-smoothed PDF weighted by the FRs at each electrode was computed. Let (*X*_*i*_, *Y*_*i*_) denote the spatial coordinates of electrode *i* and *f*_*i*_ its corresponding mean FR. For each grid point (*x*, *y*), the probability density was then defined as:

$$P_{\text{FR}}(x,y)=\frac{\sum_{i=1}^{N}f_{i}\;\exp\;\left(-\frac{(x-X_{i}{)}^{2}+(y-Y_{i}{)}^{2}}{2\sigma^{2}}\right)}{\sum_{x^{\prime },y^{\prime }}\sum_{i=1}^{N}f_{i}\;\exp\;\left(-\frac{(x^{\prime }-X_{i}{)}^{2}+(y^{\prime }-Y_{i}{)}^{2}}{2\sigma^{2}}\right)},$$

where the variance *σ* was set to 10% of the recording area. Normalization ensured that the discrete probabilities summed to one, providing a valid spatial probability distribution of firing activity.

#### Effective connectivity

To compute causal relationships among the activity of electrode pairs, a version of generalized transfer entropy (TE; Stetter et al., 2012; Orlandi et al., 2014) was run in MATLAB. The 5 min time series, originally sampled at Δ*t* = 50 μs, were binned at a resolution of 1 ms, a representative biological timescale. The results remained consistent even for larger bin choices (up to 5 ms), indicating that the strongest interactions occurred on short timescales (Timme et al., 2014). After binning, time series were binarized (“1” for the presence of a spike and “0” for its absence).

TE (Schreiber, 2000) measures the information gained (improved predictability) of the future state (*x*_*n*+1_) of a target series *X* given the state (*y*_*n*_) of a transmitting series *Y*, as:

$${\text{TE}}_{Y\to X}=\sum_{n}p(x_{n+1},x_{n}^{\left(k\right)},y_{n}^{\left(k\right)})\log\;\frac{\;p(x_{n+1}|x_{n}^{\left(k\right)},y_{n}^{\left(k\right)})}{p(x_{n+1}|x_{n}^{\left(k\right)})},$$

where *n* is a discrete time index and *k* the Markov order, set to *k* = 2. The sum goes over all possible values of *x*_*n*+1_, $x_{n}^{\left(k\right)}$, and $y_{n}^{\left(k\right)}$. To avoid spurious correlations arising from periods of synchronous bursting, a conditioning (Stetter et al., 2012) was considered, in which only bins in the data in which less than a 10 % of the network was active were used to establish effective connections. Thus, instead of just inferring functional connectivity, the aim here was to extract the pool of mono-synaptic interactions, enabling the identification of network alterations induced by the electrical stimulation.

TE was also evaluated in the conditions in which an instant feedback term was present to account for possible uncertainties associated to the spike detection of ±1 ms, but no significant differences were observed. Finally, a normalized TE was computed by dividing the raw TE value of each interaction *Y* → *X* by the entropy of the target series *X* (Timme et al., 2014):

$${\text{TE}}_{{\text{Norm}}_{Y\to X}}=\frac{{\text{TE}}_{Y\to X}}{-\sum_{n}p(x_{n})\log\;\;p(x_{n})},$$

a procedure that avoided highly active neurons to dominate the TE distribution.

Once a weighted adjacency matrix of TE scores was built, it was also converted into a two-dimensional PDF that captured the spatial density of local *EC*. The distribution was built using a Gaussian smoothing with the variance *σ* set to 10% of the recording area:

$$\begin{array}{rl} P_{\text{EC}}(x,y) & =\sum_{a_{i},b_{i}}\frac{P_{\text{dist}}(a_{i},b_{i})}{2\pi N\sigma^{2}}\\ & \;\times \exp\;\left(-\frac{(x-a_{i,x}{)}^{2}+(y-a_{i,y}{)}^{2}}{2\sigma^{2}}\right)\\ & \;\times \exp\;\left(-\frac{(x-b_{i,x}{)}^{2}+(y-b_{i,y}{)}^{2}}{2\sigma^{2}}\right), \end{array}$$

where *a*_*i*_ = (*a*_*i*,*x*_, *a*_*i*,*y*_) and *b*_*i*_ = (*b*_*i*,*x*_, *b*_*i*,*y*_) are the physical positions of interacting neurons *a* and *b* with effective connection *i*, $d_{a_{i},b_{i}}$ is the distance between those points, and *P*_dist_(*a*_*i*_, *b*_*i*_) is a weight assigned to connection *i*. This weight was set as Gaussian-dependent and modulated linearly by the connection strength, of the form:

$$P_{\text{dist}}(a_{i},b_{i})={\text{TE}}_{a_{i},b_{i}}\exp\;\left(-\frac{d_{a_{i},b_{i}}^{2}}{2\sigma^{2}}\right).$$

Both EC and FR are projected onto a 2D probability space to enable their comparison with IZs. This projection is not straightforward for the EC case, since each connection inherently links two spatial locations and thus cannot be uniquely represented by a single point in a spatial PDF. To address this difficulty, we adopted a coarse-graining approach, using Gaussian smoothing to obtain a spatially resolved representation of the connectivity weights. This procedure provides a consistent mapping of the pairwise connectivity matrix onto the same spatial scale used for the IZ distributions, allowing a direct comparison between IZ and EC.

Our analysis emphasizes local interactions, as long-range connections are effectively smoothed by the chosen coarse-graining scale and the application of distance-dependent weighting. Although long-range connection may influence and partially shape the IZs, we focus on local interactions to match the spatial scale of IZ PDF. Finally, connectivity density might not be homogeneous across the culture, but this does not constitute a limitation for the scope of the present work. It is sufficient for relatively equally dense connections to exist over an extended area. We expect then to observe shifts in IZs and connectivity between these occupied areas.

#### Centrality measures

To characterize the relative importance of nodes in the neuronal network, two complementary centrality measures were employed: betweenness centrality and spectral centrality. Betweenness centrality quantifies the extent to which a node lies on the shortest paths between other nodes in the network, capturing its role in information flow across the system (Rubinov and Sporns, 2010). On the other hand, spectral centrality corresponds to the leading eigenvector of the adjacency matrix, and assigns higher scores to nodes that are connected to other highly connected nodes (Bonacich, 1987). All centrality measures were computed on the network graph using standard definitions from network theory. Once a value was assigned to each node, a Gaussian smoothing was applied to estimate the corresponding PDF, following the procedure described in Equation 2.

#### Changes in PDFs

The difference between the probability distributions of the IZs, |Δ*P*_IZ_(*x*, *y*)|, was computed to quantify spatial changes before and after electrical stimulation. Since an IZ is represented as a spatial PDF, subtraction between phases reflects the redistribution of probability mass across space. The difference was computed as:

$$|\Delta P_{\text{IZ}}(x,y)|=\frac{\left|P_{\text{IZ}}(x,y{)}_{\text{Post\textbackslash{}, II}}-P_{\text{IZ}}(x,y{)}_{\text{Pre}}\right|}{\sum_{x,y}|P_{\text{IZ}}(x,y{)}_{\text{Post\textbackslash{}, II}}-P_{\text{IZ}}(x,y{)}_{\text{Pre}}|},$$

where the normalization bounds this quantity between 0 and 1. For both FR and EC, the subtraction was performed at the level of the raw quantities, i.e., on the FRs and on the original transfer entropy weight matrices, prior to any normalization into PDFs. This preserves the physical meaning of the differences (rate changes and connectivity weight changes) and avoids distortions that would arise from subtracting already normalized distributions. After obtaining the absolute changes, the procedure described in Sections “FR spatial distribution” and “Effective connectivity” was applied to construct the corresponding PDFs.

Similarly, in Section “Effective connectivity reveals plasticity responses,” the PDFs of weight increases and decreases were evaluated. To this end, the transfer entropy matrices of “Post II” and “Pre” were subtracted. Positive and negative components were then separated, such that the matrices ΔTE > 0 and ΔTE < 0 selectively captured strengthened and weakened connections, respectively. Each of these matrices was subsequently converted into a PDF following the previously described procedure, leading to Δ*P*_EC>0_ and Δ*P*_EC<0_, respectively.

#### Comparison of probability distributions

The PDFs of the IZs, *P*_IZ_(*x*, *y*), of the FR, *P*_FR_(*x*, *y*), and of the EC, *P*_EC_(*x*, *y*), were compared using the Jensen–Shannon divergence (JSD), a symmetric and smoothed version of the Kullback–Leibler divergence (Cover, 1999) that in a general form is given by:

$$\text{JSD}(P\|Q)=\frac{1}{2}D_{\text{KL}}(P\|M)+\frac{1}{2}D_{\text{KL}}(Q\|M),$$

where *P* and *Q* are the distributions to be compared and *M* = (1/2)(*P* + *Q*) is the mixture distribution of *P* and *Q*. JSD quantifies the similarity between two distributions, and it is bounded between 0 and 1, with 0 corresponding to identical distributions and 1 to maximally different ones. For each pairwise comparison within a recording, a single value is obtained. The extension of this procedure to all experiments provides a collection of values from which a new distribution is constructed.

Thus, for comparisons between *P*_*ξ*_(*x*, *y*) and *P*_*ξ*′_(*x*, *y*) the JSD distribution was termed *P*_*ξ*−*ξ*′_ and was constructed based on the statistics of 2(*N*_*S*_ + *N*_*C*_) = 92 observations, which correspond to “Pre” and “Post II” data for stimulated cultures, together with control recordings (two per culture). For comparisons of changes between “Pre” and “Post II” phases in *ξ* and *ξ*′, Δ*P*_*ξ*_ and Δ*P*_*ξ*′_, respectively, the new JSD distribution was termed *P*_Δ*ξ*−Δ*ξ*′_ and was based on *N*_*S*_ + *N*_*C*_ = 46 cultures.

In addition, we computed the JSD of *P*_IZ_(*x*, *y*), IZ_JSD_ between “Pre” and “Post II” to evaluate the induced changes across experimental conditions, as well as for *P*_FR_, FR_JSD_. The computed EC matrices were also compared with each other using this metric, after proper normalization, yielding EC_JSD_. It should be noted that the use of Gaussian smoothing and the subsequent comparison using JSD addresses the issue of fluctuations by treating them as background noise.

Other simple metrics, such as the Euclidean distance, were discarded given the weighted nature of the networks (Martínez and Chavez, 2019) as well as the strong fluctuations in *P*_EC_(*x*, *y*) associated to non-significant connections. Although these additional metrics were tested, they did not yield conclusive results.

#### Statistical analysis

Statistical and graphical analyses were carried out using MATLAB 2024a. Mann–Whitney *U* tests were used to analyze the following quantities: (1) *ξ*_JSD_ between the “Control” and “Stim” experimental conditions, and (2) the difference in the mean FR and the mean IBI between the same conditions. Two-sample Kolmogorov–Smirnov tests were used to analyze the following: (1) the JSD differences *P*_*ξ*−*ξ*′_ compared to random pairing, (2) the JSD differences of distribution changes *P*_Δ*ξ*−Δ*ξ*′_ versus random pairing, and (3) the JSD between the increase and the decrease of *P*_EC_(*x*, *y*) versus random pairing. One sample Student’s *t*-tests were performed to evaluate whether the distribution of FR differences and IBI differences significantly differed from zero. Statistical significance was set at *p* < 0.05 for all analyses. When appropriate, data were represented and examined via boxplots and via Kernel density estimators (KDE).

## Results

### Alteration of burst IZs upon stimulation

**Figure 4.**
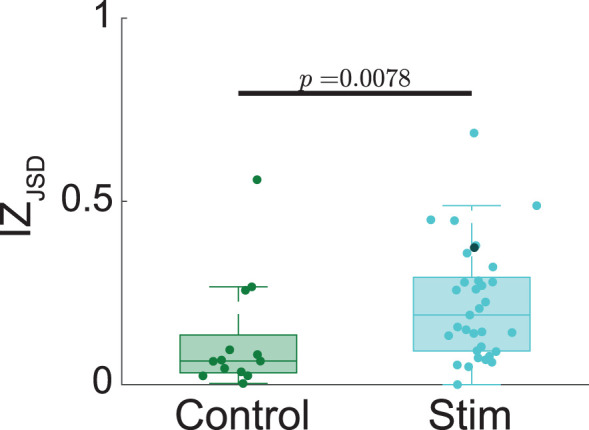
Distribution of JSD values for IZs between the “Pre” and “Post II” phases in a given experiment, comparing controls and stimulated cultures. The corresponding analyses of JSD values between the “Pre” and “Post I” and between “Post I” and “Post II” phases are shown in Figure 4-1, whereas comparisons across consecutive DIV are provided in Figure 4-2. In the panel shown here, each dot represents a neuronal culture, with the representative experiment from Figure 6*A* highlighted in a darker tone. The JSD is significantly higher in stimulated cultures compared to controls (*U* = 105, *p* = 0.008, *N*_Ctrl_ = 13, *N*_stim_ = 33, Mann–Whitney *U* test). Related to this analysis, the variability in IZs activation showed no significant correlation with the magnitude of the IZ shift (Fig. 4-3).

We investigated the response of in vitro cortical neuronal cultures to electrical stimulation. Cultures were grown on HD-MEAs, which were used to both record spontaneous activity and to deliver a stimulation protocol (Fig. 1 and Section “Materials and Methods”). We focused the analysis on the “Pre” and “Post II” phases, i.e., just before stimulation and 1 h after it, to compare the basal state of the neuronal culture (“Pre”) with the effects of an electrical perturbation on a long timescale (“Post II”). The corresponding comparisons between “Pre” and “Post I,” and between “Post I,” and “Post II” are provided in the Extended Data (Fig. 4-1). Our goal was to identify long-term changes in activity following electrical perturbation and to relate these to possible structural changes due to network connectivity remodeling.

Since neuronal network dynamics are our direct observable, we first investigated whether network-wide dynamics in the form of bursting events serve as a signature to detect large-scale alterations due to stimulation. For that, we focus on *burst IZs*, the regions in the physical space of the neuronal culture where bursts nucleate, given their high sensitivity to network connectivity (Orlandi et al., 2013). These IZs are specific to each culture and are shaped through a complex phenomenon governed by the non-trivial interplay between dynamics and topology. Figure 2*A* provides an example of spontaneous activity that highlights the presence of network bursts, which appear as apparent vertical lines indicating strong coherent activation of the network. A closer inspection of the first burst (Fig. 2*B*) shows that neuronal activation throughout the network occurs in a progressive manner in a timespan of about 50 ms, with few neurons leading activity initiation and then triggering activation of the neighboring ones in a cascading manner.

The snapshots of network activity evolution in the culture at different time windows (Fig. 2*B*,*C*) reveals a wave-like propagation of activity, initiating on the top-right corner and then gradually advancing leftwards. This analysis, extended to all bursts, provided the set of burst initiation points of the recording, which was transformed into the PDF of observing burst initiation in an area of the culture, *P*_IZ_(*x*, *y*). Its maxima correspond to the IZs.

As shown in Figure 3*A*, left, the distribution of burst IZs along a recording was strongly concentrated, with the nucleation probability confined to a tight region in the top-right corner. When the culture was left unperturbed and measured again 1 h later (control condition), the burst IZs remained in the same location (Fig. 3*A*, right), indicating that, at least on timescales of hours, burst initiation was spatially stable.

In contrast, a markedly different behavior was observed in a separate culture subjected to electrical stimulation, shown in Figure 3*B*, and where the red squares on the images mark the stimulated electrodes. Initiation probability before stimulation (Fig. 3*B*, left) was concentrated in the bottom-left corner. However, 1 h after stimulation, this probability shifted to the bottom-right corner of the culture (Fig. 3*B*, right), revealing a remodeling of the network’s spontaneous activity.

We note that multiple IZs are possible in the same neuronal culture, i.e., regions that display comparable initiation probabilities. The way these regions interact can be qualitatively described in the context of the work of Feinerman et al. (2007), which proposed a boxing model to understand the interaction of burst initiation sites in 1D cultures. In that model, each initiation region has a certain firing frequency and competes to ignite the burst.

Our results can also be understood within this framework. In Figure 3*B*, for instance, a single zone dominates initiation dynamics before stimulation, whereas after stimulation two zones coexist, one on the bottom-right (with 45% of initiation events) and one on the bottom-left (25% of initiation events). The intriguing aspect, however, is the change in spatial localization of burst IZs as stimulation takes action.

The transition observed in Figure 3*B* was progressive along the 60 min window after electrical stimulation ended. As illustrated in Figure 3*C* for a different culture, the shift did not involve a sudden migration toward a newly generated region, but rather a gradual transition from the basal to the stimulation-remodeled basin of attraction.

To summarize the impact of stimulation on the different experimental realizations, we analyzed the distribution of JSD values for the IZs between the “Pre” and “Post II” phases (IZ_JSD_, Fig. 4), comparing control cultures to stimulated ones. Stimulated cultures exhibited significantly higher JSD values as compared to controls (*U* = 105, *p* = 0.008, *N*_Ctrl_ = 13, *N*_stim_ = 33, Mann–Whitney *U* test), indicating that the electrical perturbation substantially shifts the spatial distribution of burst IZs. Examination of the timing of this shift between phases revealed that the changes to IZs were not instantaneous, but occurred over a period of around 1 h, since no significant difference between “Pre” and “Post I” was found, but the significant difference appeared between “Post I” and “Post II” (Fig. 4-1). This indicates that stimulation did not induce a transitory change following a return to the original state, but kicked off a drift consistent with the aforementioned gradual transition. Finally, the variability of IZ activation—understood as the degree to which initiation events are distributed across different locations during a single recording, quantified by the Gini coefficient of the initiation probability distribution—showed no significant correlation with the magnitude of the IZ shift (Fig. 4-3). This indicates that the stimulation-induced shift in IZ location is largely independent of how spatially distributed or concentrated the IZs are.

10.1523/ENEURO.0457-25.2026.f4-1 Figure 4-1 Changes in IZ, EC, and FR between the‘Pre’ and ‘Post I’ phases and between the ‘Post I’ and ‘Post II’ ones. **(A)** Distribution of JSD values for IZs (left), EC (middle) and FR (right) between ‘Pre’ and ‘Post I’, comparing controls and stimulated cultures. Each dot represents a neuronal culture, with the representative experiment from Fig. 6A highlighted in a darker tone. No significant differences are observed in the JSD between control and stimulated conditions for none of the measures (IZ: *U* = 56, *p* = 0.16; EC: *U* = 85, *p* = 0.85; FR: *U* = 73, *p* = 0.23; *N_Ctrl_* = 6, *N_stim_* = 30, Mann–Whitney U test) **(B)** Corresponding data for the ‘Post I’ and ‘Post II’ phases, comparing controls and stimulated cultures. Each dot represents a neuronal culture, with the representative experiment from Fig. 6A highlighted in a darker tone. IZJSD is significantly higher in stimulated cultures compared to controls (*U* = 42, *p* = 0.044, *N_Ctrl_* = 6, *N_stim_* = 30, Mann–Whitney U test), whereas no significant differences are observed for either ECJSD (*U* = 76, *p* = 0.57, *N_Ctrl_* = 6, *N_stim_* = 30, Mann–Whitney U test) or FRJSD: (*U* = 87, *p* = 0.91, *N_Ctrl_* = 6, *N_stim_* = 30, Mann–Whitney U test). Download Figure 4-1, TIF file.

10.1523/ENEURO.0457-25.2026.f4-2 Figure 4-2 Burst IZs shift across consecutive days. **(A)** Relationship between the IZ*_JSD_* of two consecutive days for the ‘Pre’ phases *JSD*(*Pre_i_*, *Pre_i_*_+1_), and the IZ*_JSD_* between the ‘Post II’ and next ‘Pre’ phase, *JSD*(*Pos*tII*_i_*, *Pre_i_*_+1_). The dashed line indicates equality. Points laying below the diagonal indicate experiments in which the IZ configuration wasmaintained after the stimulation-induced shift, whereas points above the diagonal indicate cultures that returned to their original IZ configuration after one day. Color indicates the IZ*_JSD_* between the ‘Pre’ and the ‘Post II’ phase of the same day. No clear trend for maintenance or recovery is observed. **(B)** Retention index, defined as *JSD*(*Pre_i_*, *Pre_i_*_+1_) − *JSD*(*Post*II*_i_*, *Pre_i_*_+1_), comparing the similarity between consecutive ‘Pre’ IZ distributions and the similarity between ‘Post II’and subsequent ‘Pre’ epochs. The observed distribution was compared against a randomized distribution, showing no significant difference (*D* = 0.19, *p* = 0.49, *N* = 18, *N_rand_* = 5000, Kolmogorov-Smirnov test). Download Figure 4-2, TIF file.

10.1523/ENEURO.0457-25.2026.f4-3 Figure 4-3 IZJSD between the ‘Pre’ and ‘Post II’ phases for the stimulated condition as a function of the Gini coefficient of the IZ spatial probability distribution. The Gini coefficient quantifies heterogeneity, with 0 corresponding to uniformly distributed initiation spots and 1 indicating complete concentration at a single site. The blue dots in the plot represent the studied cultures. The Pearson correlation between the two measures (red line) is not significant (ρ = 0.3, *p* = 0.09). Download Figure 4-3, TIF file.

### EC captures IZs displacement

We used EC inferred through TE to quantify neuronal network communication before and after stimulation. As detailed in the Methods section, EC was computed from time series of neuronal activity data, procuring a weighted matrix containing the strength of effective connections among all electrode pairs. We interpret stimulation-induced changes in EC as a proxy for structural synaptic modifications driven by plasticity (Butz et al., 2009; Yildirim-Keles et al., 2025). With the aim to reveal changes in synaptic strength that can be later compared with shifts in burst IZs, we transformed the weighted adjacency matrix into a PDF *P*_EC_(*x*, *y*).

**Figure 5.**
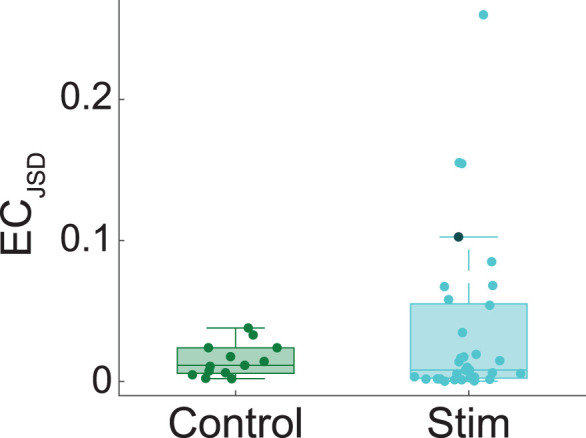
Distribution of JSD values for EC between the “Pre” and “Post II” phases in a given experiment, comparing controls and stimulated cultures. Each dot represents a neuronal culture, with the representative experiment from Figure 6*A* highlighted in a darker tone. No significant differences are observed in the JSD between control and stimulated conditions (*U* = 201, *p* = 0.7, *N*_Ctrl_ = 13,*N*_stim_ = 33, Mann–Whitney *U* test).

Once we had PDFs, we were able to compute EC_JSD_ to compare the JSD values for the ECs between the “Pre” and “Post II” phases. JSD analysis for the EC (Fig. 5) showed no significant difference between control and stimulated cultures (*U* = 201, *p* = 0.7, *N*_Ctrl_ = 13,*N*_stim_ = 33, Mann–Whitney *U* test).

The discrepancy between the pronounced IZs displacement and the modest EC shift indicates that subtle changes in neuronal coupling driven by stimulation are enough to reshape activity propagation. Such changes can modify the amplification pathways of network activity and thus alter the relative dominance of different IZs. In consequence, IZs prove to be a good indicator to alterations promoted by stimulation. In contrast, EC captures changes in neuronal interactions that do not necessarily originate from the electrical perturbation, but that may include network-wide homeostatic responses, overall smoothing out signatures of network reconfiguration. We remark that EC by itself is not sufficiently sensitive to detect network remodeling, and that substantial alterations in EC are not needed to observe changes in the spatial structure of spontaneous activity, which are properly captured by IZs variation. However, despite its limited sensitivity to stimulation-induced remodeling, EC still offers meaningful insight into overall network connectivity, thereby supporting the interpretation of IZ changes even in the absence of significant EC alterations.

**Figure 6.**
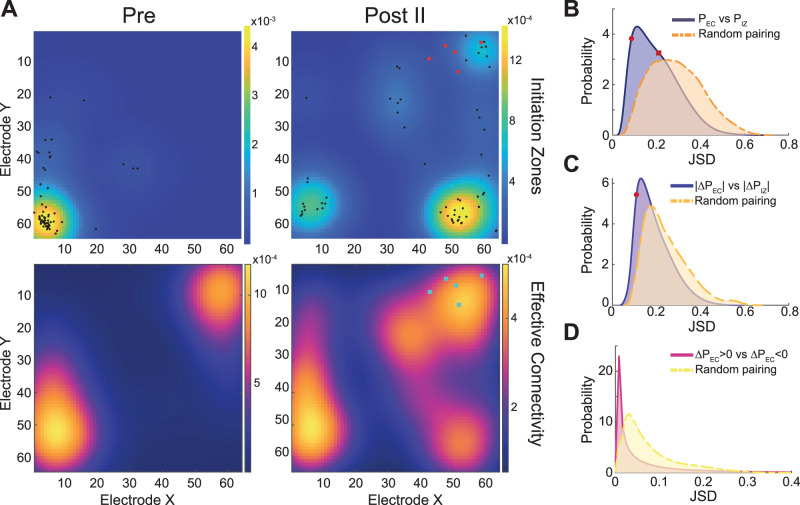
Comparison between IZs and EC. ***A***, PDFs of IZs, *P*_IZ_(*x*, *y*) (top), and EC, *P*_EC_(*x*, *y*) (bottom), comparing the scenarios before stimulation (left) and 1 h after it (right). Stimulated electrodes are located in the top right corner and are marked as red or blue squares. Both maps reveal stimulation-induced changes, particularly in the lower half of the culture, with similar but not fully overlapping patterns. This representative example, used throughout the article, corresponds to Batch 1, DIV24, Label 722. ***B***, Kernel density estimation (KDE) of the Jensen–Shannon divergence (JSD) between *P*_EC_(*x*, *y*) and *P*_IZ_(*x*, *y*) (purple curve), compared with to JSD values from randomized pairs (orange). The divergence is significantly lower in the non-random case (*D* = 0.34, *p* = 6 · 10^−13^, *N* = 128, *N*_rand_ = 5,000, Kolmogorov–Smirnov test), indicating statistical correspondence between the two distributions. Square and circle markers denote the JSD values for the “Pre” and “Post II” phases of the experiment shown in panel ***A***, respectively. JSD was also evaluated between *P*_IZ_ and *P*_*BC*_ and between *P*_IZ_ and *P*_*SC*_, the betweenness and the spectral centrality, respectively (Fig. 6-1). ***C***, KDE of the JSD between the absolute changes in EC and IZs, |Δ*P*_EC_(*x*, *y*)| and |Δ*P*_IZ_(*x*, *y*)| (purple), compared to randomized pairs (orange). Distributions are significantly different (*D* = 0.27, *p* = 6 · 10^−8^, *N* = 118, *N*_rand_ = 5,000, Kolmogorov–Smirnov test), suggesting coordinated changes in response to stimulation. ***D***, KDE of the JSD of Δ*P*_EC_(*x*, *y*) for regions showing increases versus decreases in EC (pink), compared to randomized pairs (yellow). Distributions are significantly different (*D* = 0.56, *p* = 3 · 10^−10^, *N* = 33, *N*_rand_ = 5,000, Kolmogorov–Smirnov test).

As shown in Figure 6*A*, which considers the same dataset as in Figure 3*B*, the EC partially captures the distribution of the IZs, with clusters of strongly connected electrodes aligning well with prominent IZs.

10.1523/ENEURO.0457-25.2026.f6-1 Figure 6-1 Spatial correspondence between centrality measures and burst initiation zones. **(A)** KDE of the JSD between the smoothed betweenness centrality PDF, *P*_BC_(*x*, *y*) and *P*_IZ_(*x*, *y*) (purple curve), compared with to JSD values from randomized pairs (orange). The divergence is significantly lower in the nonrandom case (*D* = 0.18, *p* = 8·10^−4^, N = 128, *N_rand_* = 5000, Kolmogorov-Smirnov test), indicating statistical correspondence between the two distributions. **(B)** KDE of the JSD between the smoothed spectral centrality PDF, *P*_SC_(*x*, *y*) and *P_IZ_*(*x*, *y*) (purple curve), compared with to JSD values from randomized pairs (orange). The divergence is significantly lower in the non-random case (*D* = 0.29, p = 2·10^−9^, N = 128, *N_rand_* = 5000, Kolmogorov-Smirnov test), indicating statistical correspondence between the two distributions. Download Figure 6-1, TIF file.

To quantify the similarity between the spatial distribution of IZs and EC, we computed the JSD between *P*_IZ_(*x*, *y*) and *P*_EC_(*x*, *y*) upon stimulation. Figure 6*B* shows in purple the JSD distribution that compares the IZs and TE distributions, *P*_IZ-*TE*;_, for every phase in every condition (92 experimental recordings in total). The JSD curve is strongly peaked at low values, indicating that the spatial arrangements of IZs and EC are very similar. The specific JSD values of the “Pre” and “Post II” data of Figure 6*A* are highlighted with a square and a circle symbol, respectively, to indicate their position within the distribution. To determine whether the JSD values obtained were due to the overlap between the specific *P*_IZ_ and *P*_EC_ of a single recording, and not due to the nature of the data, we compared the *P*_IZ-*TE*;_ JSD distribution with the one obtained for a random pairing of connectivity and initiation maps (orange curve). The distribution of JSD of randomly paired IZs and EC PDFs differed significantly (*D* = 0.34, *p* = 6 · 10^−13^, *N* = 128, *N*_rand_ = 5,000, Kolmogorov–Smirnov test) from the matched distribution. The random pairing distribution is broader and contains higher JSD values, hence indicating larger divergence.

Although the JSD curves tended to be concentrated toward low values, they were far from being sharply peaked at 0. This means that the correlation between IZs and ECs was only partial, a result that was also expected given the fact that clusters of strong connectivity are a necessary but not sufficient condition to ignite a burst. Indeed, the studies of Orlandi et al. (2013) and Orlandi and Casademunt (2017) showed, using numerical simulations, that IZs correlate with the ignition avalanches field, which is constituted by the activity of neurons being activated prior to the burst (Tajima et al., 2017), but not by the structural connectivity traits of the neuronal network. In addition, experimental work has shown that regions with particularly high or low activity, a characteristic influenced by recurrent connectivity, are less likely to act as burst IZs, possibly due to synaptic short-term depression following repeated recruitment during bursts (Okujeni and Egert, 2019). Finally, activity originated outside the electrodes recording area might skew the IZs PDF toward the boundaries, while EC is obtained just from the recording area.

To further characterize the structural role of different regions in the inferred connectivity network, we computed additional graph-theoretic centrality measures, namely betweenness and spectral centrality. Similarly to EC, the spatial distributions of these metrics did not reveal a systematic correspondence with burst IZs beyond a partial overlap (Fig. 6-1). Nevertheless, there was also a significant difference compared with random pairing, which was more evident for spectral centrality.

Given that burst IZ distributions shift following stimulation, and that IZs and EC maps exhibit spatial overlap, we next asked whether changes in IZ distribution and changes in the EC distributions were spatially correlated. We next compared the absolute changes in the weights of effectively connected neurons from “Pre” and “Post II” phases (|Δ*P*_EC_|) with the corresponding changes in the IZs (|Δ*P*_IZ_|). The comparison was quantified using the JSD of |Δ*P*_IZ_| and |Δ*P*_EC_| (Fig. 6*C*, purple curve), which was again compared with random pairing (Fig. 6*C*, orange curve). The JSD curves are based on 46 experimental recordings (a combination of the “Pre” and “Post II” phases constitutes one recording). Experiment-matched data and random paired experiments were significantly different (*D* = 0.27, *p* = 6 · 10^−8^, *N* = 118, *N*_rand_ = 5,000, Kolmogorov–Smirnov test), indicating that changes in EC correlate spatially with changes in the distribution of burst initiation.

It is important to emphasize the use of absolute changes, since an increase in effective connections within a network region alone does not necessarily enhance its capacity to trigger bursts. In other words, we focus on the magnitude of the association between alterations in EC and IZ, irrespective of whether they occur in the same or opposite directions.

### EC reveals plasticity responses

IZs emerge from a complex interplay of neuronal dynamics, short- and long-range connections within the network, and intrinsic noise. As a result, IZs do not provide direct local spatial information about synaptic remodeling driven by plasticity. In contrast, EC analysis—based on pairwise interactions of spatially-localized neurons—offers spatial insights into changes in neuronal communication that can be interpreted as synaptic alterations. Therefore, EC can be employed to investigate the plasticity mechanisms underlying the neuronal culture’s response to electrical perturbation. To that aim, we investigated the regions within the culture that exhibited the most pronounced changes in EC.

The regions exhibiting the largest increases in effective weights, i.e., the maxima of Δ*P*_EC>0_, substantially overlapped with those showing the strongest decreases, the maxima of Δ*P*_EC<0_ (Fig. 6*D*). This overlap was quantified using the JSD between the positive and negative increment of the smoothed EC map. The resulting distribution (Fig. 6*D*, pink curve) sharply peaked around zero, indicating a near-complete spatial correspondence between potentiation and de-potentiation. This distribution was significantly different (*D* = 0.56, *p* = 3 · 10^−10^, *N* = 33, *N*_rand_ = 5,000, Kolmogorov–Smirnov test) from the random pairing (Fig. 6*D*, yellow curve), although the latter also provided a relatively low JSD value due to the overall spatial homogeneity of the connectivity changes.

Given the pronounced overlap between regions exhibiting the largest increases and decreases in EC, we interpreted these changes as indicative of synaptic remodeling rather than a uniform strengthening of connections. This correspondence is consistent with the involvement of homeostatic plasticity mechanisms, which are known to activate compensatory adjustments to stabilize network activity (Lu et al., 2025). In the absence of a stimulation protocol with local synaptic specificity, the network appeared to reorganize its connectivity in response to the external perturbation, balancing potentiation and de-potentiation to maintain overall functional stability.

### FR does not reflect IZs shift

Besides IZs, an important observable related to the network activity is the FR of the individual neurons or electrodes. In this section we investigate the effect of the electrical stimulation on the FRs, and the link between the spatial distribution of FRs, and the spatial distribution of IZs and EC.

**Figure 7.**
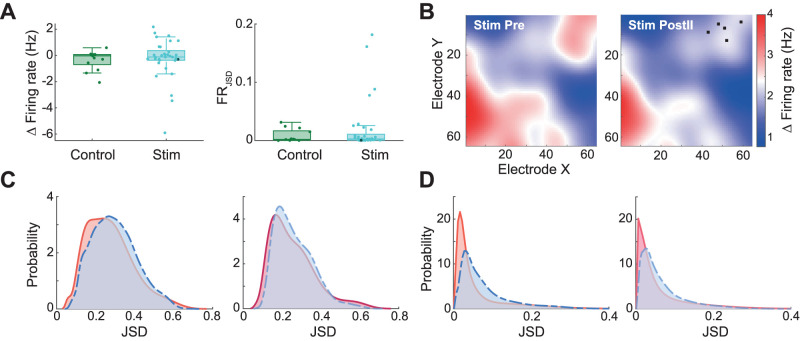
Firing rates (FRs) and effective connectivity. ***A***, Box plots of the differences in network-averaged FRs (left) and corresponding Jensen–Shannon divergence FR_JSD_ (right) between “Pre” and “Post II” phases for control and stimulated cultures. No significant differences are observed between conditions, neither for FRs (*U* = 172, *p* = 0.25, *N*_Ctrl_ = 13, *N*_Stim_ = 33, Mann–Whitney *U* test) nor for FR_JSD_ (*U* = 194, *p* = 0.63, Mann–Whitney *U* test). The FR difference distributions are centered around zero, indicating that stimulation does not substantially alter the intrinsic dynamics of the neuronal cultures (Control: *t* = −0.8, *p* = 0.1, *N*_Ctrl_ = 13; Stim: *t* = −0.8, *p* = 0.8, *N*_Stim_ = 33, Student’s *t*-test). The differences in network-averaged firing rates between the “Pre” and “Post I” and between “Post I” and “Post II” phases are provided in Figure 7-1. ***B***, Gaussian-smoothed spatial maps of FRs for the stimulated culture representative example, comparing the “Pre” (left) and “Post II” (right) phases. ***C***, Left: KDE of the JSD between *P*_FR_(*x*, *y*) and *P*_IZ_(*x*, *y*) (red), compared to randomized pairs (blue). Distributions are not significantly different (*D* = 0.11, *p* = 0.07, *N* = 128, *N*_rand_ = 5,000, Kolmogorov–Smirnov test). Right: KDE of the JSD between the absolute changes in FR and IZs, |Δ*P*_FR_(*x*, *y*)| and |Δ*P*_IZ_(*x*, *y*)| (red), compared to randomized pairs (blue). Distributions are not significantly different (*D* = 0.10, *p* = 0.23, *N* = 118, *N*_rand_ = 5,000, Kolmogorov–Smirnov test). ***D***, Left: KDE of the JSD between *P*_FR_(*x*, *y*) and *P*_EC_(*x*, *y*) (red), compared to randomized pairs (blue). Distributions are significantly different (*D* = 0.34, *p* = 3 · 10^−13^, *N* = 128, *N*_rand_ = 5,000, Kolmogorov–Smirnov test). Right: KDE of the JSD between the absolute changes in FR and EC, |Δ*P*_FR_(*x*, *y*)| and |Δ*P*_EC_(*x*, *y*)| (red), compared to randomized pairs (blue). Distributions are significantly different (*D* = 0.19, *p* = 3 · 10^−4^, *N* = 118, *N*_rand_ = 5,000, Kolmogorov–Smirnov test).

For each activity measure, we quantified its changes by comparing the variation between the “Pre” and “Post II” phases in stimulated cultures, and between two recordings spaced 1 h apart in control cultures. FR did not significantly differ between stimulated and control conditions (Fig. 7*A*, left). This does not mean that FRs remained constant at every electrode. Rather, individual electrodes exhibited both increases and decreases in activity, which canceled out when averaged across the culture.

10.1523/ENEURO.0457-25.2026.f7-1 Figure 7-1 Boxplots of the differences in network-averaged firing rates between ‘Pre’ and ‘Post I’ (left) and ‘Post I’ and ‘Post II’ (right). No significant differences are observed between conditions (Pre vs PostI: *U* = 89, *p* = 0.98; Post I vs Post II: *U* = 73, *p* = 0.5; *N_Ctrl_* = 6, *N_Stim_* = 30, Mann-Whitney-U test). Download Figure 7-1, TIF file.

Additionally, a Student’s *t-*test showed that the corresponding distributions did not significantly deviate from zero. To further assess the temporal stability of firing activity, we compared FRs across all experimental phases (“Pre,” “Post I,” and “Post II”), finding no significant differences between successive stages (Fig. 7-1). While stability in FRs is expected in controls, its persistence in stimulated cultures indicates that the observed reorganization of burst IZs and EC occurs without disrupting overall network activity, possibly reflecting the influence of homeostatic mechanisms.

It is worth to restate that this analysis of changes in FR is based on average values of activity across entire cultures. A complementary approach involves analyzing electrode-level statistics to assess whether stimulation induces localized changes, such as increased dispersion in activity levels or significantly altered IBI distributions. Following the same pipeline used with IZs and EC, we computed the Gaussian-smoothed PDF of FRs to account for spatial information, *P*_FR_(*x*, *y*). Despite having the FRs at a higher spatial resolution, the smoothing enables a meaningful comparison with the distributions *P*_IZ_(*x*, *y*) and *P*_EC_(*x*, *y*), since it captures trends at a mesoscopic scale. When comparing the resulting JSD curve for FRs between stimulated and control cultures (termed FR_JSD_), we did not observe significant differences (Fig. 7*A*, right). This indicates that local electrical stimulation did not elicit a detectable spatial reorganization of the FR smoothed PDF.

We also examined the raw FR maps for stimulated cultures during the “Pre” (Fig. 7*B*, left) and “Post II” (Fig. 7*B*, right) phases for the representative example of an stimulated case. Unlike the probability density representations *P*_IZ_(*x*, *y*) and *P*_EC_(*x*, *y*), these maps display the actual FRs recorded at each electrode. Qualitatively, the spatial pattern of FRs appeared more consistent with the EC distribution than with the IZs distribution, suggesting that spontaneous network activity may be more strongly aligned with the endogenous connectivity configuration than with the imposed stimulation site. Indeed, the bottom-right corner of the culture exhibited a prominent hotspot in the IZ maps, which corresponds to an area of relatively weak or moderate activity in the FR map. This strong discrepancy can be interpreted through the noise focusing framework, which argues that local increases in FRs are not a sufficient ingredient to initiate bursts. Instead, the key factors lie in the network’s topological architecture and dynamic properties, which ultimately govern the propagation and convergence of activity toward basins of attraction.

To further quantify possible relationships between spatial firing rates and IZ distribution, we computed the spatial overlap between the FR maps, *P*_FR_(*x*, *y*), with *P*_IZ_(*x*, *y*) using JSD (Fig. 7*C*, left, red curve) and compared this distribution to the one obtained from random pairings (Fig. 7*C*, left, blue curve). These two distributions did not differ significantly (*D* = 0.11, *p* = 0.07, *N* = 128, *N*_rand_ = 5,000, Kolmogorov–Smirnov test), indicating that IZs do not correlate with high FR areas of the culture (Okujeni and Egert, 2019). To further discard any possible relation between these two measures, we also quantified the spatial correlation of their changes, |Δ*P*_FR_| and |Δ*P*_IZ_|, (Fig. 7*C*, right). Despite peaking at a lower value than *P*_FR-_*IZ*;, there was no significant difference with the random pairing (*D* = 0.10, *p* = 0.23, *N* = 118, *N*_rand_ = 5,000, Kolmogorov–Smirnov test). Therefore, the changes in the FR are not correlated with the IZs shift.

We next assessed the relationship between *P*_FR_(*x*, *y*) and *P*_EC_(*x*, *y*) (Fig. 7*D*, left). The JSD between these two distributions was significantly lower than that obtained from random pairings (*D* = 0.34, *p* = 3 · 10^−13^, *N* = 128, *N*_rand_ = 5,000, Kolmogorov–Smirnov test). This overlap is expected, given that EC is inferred from the time series, and FR is the electrode-average of those time series. To examine whether FR variations reflect changes in connectivity, we compared the spatial changes |Δ*P*_EC_| and |Δ*P*_FR_| using the same JSD-based approach (Fig. 7*D*, right). In this case, the JSD also significantly differed from random pairings (*D* = 0.19, *p* = 3 · 10^−4^, *N* = 118, *N*_rand_ = 5,000), although the Kolmogorov–Smirnov statistic, *D*, is much smaller. Hence, while FR and EC share a similar spatial organization, fluctuations in FR are more weakly correlated with alterations in EC.

## Discussion

In this work, we studied the impact of electrical stimulation on the spontaneous activity of in vitro cortical neuronal networks cultured on HD-MEAs. We investigated the effects of the electrical perturbation using burst’s initiation statistics, and observed a significant reorganization of burst IZs. This indicates that IZs by themselves can be used as a markers for quantifying network alterations driven by external perturbations. To shed light on the mechanisms underlying this reorganization in network bursting dynamics, we examined the local density of effective connections through TE, which provides an indirect measure of changes in synaptic weights. We observed that IZs displacement and EC variations exhibited some degree of similarity in their spatial patterning after electrical stimulation. However, while EC changes may contribute to explaining IZs alterations, they lacked the sensitivity needed to capture the full extent of network remodeling. Below, we elaborate on the reproducibility of the results, their interpretation, as well as future directions of the devised experiments and analysis tools.

### Reproducibility and control of IZs displacement

The HD-MEA allows control over both the number and spatial arrangement of stimulated electrodes. In exploratory tests, we implemented two generic stimulation configurations: multiple electrode pairs distributed across the recording area, and compact groups of 4–5 electrodes localized within a small region. However, these configurations were not explored systematically across experiments, and therefore no quantitative comparison between them is presented here. Similarly, although in some experiments stimulation targeted regions close to the dominant IZ qualitatively identified during the “Pre” phase, the experiments were not designed to formally compare stimulation within or outside IZs. Accordingly, the present study focuses on the overall effects of stimulation on IZ and EC organization rather than on the dependence of these effects on the spatial configuration of stimulation, which can be explored in future works.

In some cultures, the primary IZ shifted to an apparently new random location, while in others it remained stable at its original position. Those cultures in which the dominant IZ remained unchanged correspond to data points closest to zero in Figure 4. The stability observed under this stimulation scheme of targeting the dominant IZ can be understood in the context of the work by Massobrio et al. (2015), who reported that synapses in dissociated neuronal cultures may become saturated due to the high density of established connections, resulting in a network that is highly stable from a dynamic point of view. Nevertheless, disparities across cultures prepared under nearly identical conditions highlight their intrinsic capacity to diverge over time, potentially reflecting the amplification of subtle mesoscale inhomogeneities (Okujeni and Egert, 2019). These inhomogeneities affect the network’s dynamic state, for example by modulating the propensity for super- or subcritical activity, which in turn influences network recruitment (Okujeni and Egert, 2023).

It is worth noting that spatial inhomogeneities consistently emerge in a similar configuration across multiple cultures. In both IZs and EC, maxima tend to appear near the corners of the electrode array. This is the reason why even randomly paired JSD distributions are more populated toward low values. This cross-culture similarity may arise from geometrical factors. This phenomenon has been previously reported by Okujeni and Egert (2019) who attribute it to frontier effects that locally enhance neuronal connectivity. On the other hand, geometry of the enclosure alone has been shown to trigger network-wide extreme events in other complex systems, such as schooling fish (Puy et al., 2024), independent of connectivity strengthening.

The clear uncontrollability of the network’s response to stimulation remains intriguing. The location of electrical stimulation does not influence the future position of IZs. This lack of controllability may be attributed to the low-frequency component of the stimulation protocol, which was shown to alter connectivity in an unpredictable manner (Le Feber et al., 2010). Indeed, Vajda et al. (2008) proposed three potential mechanisms to explain changes in network connectivity following low-frequency stimulation: (i) synaptic plasticity, (ii) modulation of intrinsic neuronal properties, and (iii) transitions between attractor states. While our study primarily focuses on plasticity, the other two mechanisms offer complementary insights into the capacity of an external stimulation to shape network dynamics. Furthermore, we remark that low-frequency stimulation protocols have been shown not to significantly affect global network properties, such as mean synaptic strength (Le Feber et al., 2010).

With the above spectrum of experimental observations at hand, our interpretation of the network’s behavior to stimulation aligns with the framework proposed by Panas et al. (2015), who suggested that spontaneous activity in neuronal cultures exhibits characteristics of a sloppy behavior, where most parameters—such as excitability and pairwise connection strength—can vary substantially without inducing global changes. This balance between robustness and evolvability allows the network to maintain global stability through homeostatic regulation of only a few key parameters. Their findings indicate that neurons with high firing rates are more sensitive to perturbations than clusters of strong functionally connected neurons. Additionally, neurons with high background conductance, especially those grown on MEAs, tend to exhibit weaker LTP (Delgado et al., 2010), which may explain our difficulty in interpreting our observations within such plasticity mechanism, as neurons may be less responsive to stimulation-induced changes. This interpretation is consistent with synaptic scaling, wherein individual synaptic weights and excitability levels are allowed to fluctuate, while their overall distribution remains stable (Statman et al., 2014).

In light of the above considerations and our results, we hypothesize that homeostatic plasticity (Lakhani et al., 2024) predominates over other mechanisms, balancing neuronal network activity by allowing the neurons to adapt their intrinsic excitability and synaptic responses according to the intensity of the inputs they experience. These homeostatic changes, eventually, prevent the network from becoming hypo- or hyperactive. Because stimulation was delivered independently of the instantaneous network state, it sampled different phases of activity. As phase-dependent stimulation can induce either potentiation or depression (Stegenga et al., 2010), the observed effects likely reflect the net outcome of these competing mechanisms. Overall, our work suggests that the network adapts to external perturbations in an attempt to maintain stable network-wide dynamics. Indeed, Po et al. (2025) applied a dimensionality reduction analysis on the same dataset presented here and observed how the “Post II” phase was closer to “Pre” than to “Post I.”

Although the electrical stimulation protocol was designed to induce LTP on specific connections (Jimbo et al., 1999), our analysis did not reveal spatially restricted potentiation, consistent with previous studies which rejected this hypothesis of a “locally-confined synaptic enhancement” (Bonhoeffer et al., 1989). Because extracellular stimulation can directly activate axonal and dendritic processes, evoked action potentials may originate in neurites passing near the stimulation electrode and propagate both orthodromically and antidromically, so that the somata of activated neurons may lie at a distance from the stimulation site. Conversely, extracellular recordings are biased toward neurons whose somata or axon initial segments are close to the electrodes, as spike amplitudes decay with distance (Pettersen et al., 2012; Radivojevic et al., 2016; Bakkum et al., 2019). As a result, the population of neurons recorded at a given location does not necessarily coincide with the population directly activated by stimulation, which complicates a direct spatial correspondence between stimulation sites and IZs. This further supports our focus on network-level effects rather than strictly local spatial relationships. Although no significant changes were observed when possible plasticity effects were examined at a network level (Chiappalone et al., 2008), we identified markers—specifically EC—that serve as proxies for underlying structural changes upon remodeling, but we were unable to pinpoint specific synaptic-level plasticity mechanisms.

### Noise focusing and the boxing model

Our results are built upon the noise focusing framework, which posits that a strong spatiotemporal localization of noise triggers bursts of spontaneous activity. In this context, IZs act as sinks that accumulate and amplify noise, acting as basins of attraction. However, since IZs emerge from network-wide, non-local mechanisms (Orlandi et al., 2013), their spatial distribution does not correlate with local network properties or other observables related to neuronal dynamics. This is illustrated in Figure 6*A*, where local EC only partially captures IZs distribution. Thus, a network may contain regions of strongly connected neurons that do not necessarily initiate bursts. For instance, by examining in detail the top right corner of the “Pre” phase in Figure 6*A*, we observed that such spot was characterized by neurons with high firing rates together with a local connectivity characterized by a large betweenness centrality—a hallmark of a strong flow of information—yet no bursts originated from this area.

The predominance of focused IZs can be understood within the “boxing model” (Feinerman et al., 2007), which proposes that different regions of the culture spontaneously activate at contrasting rates, with these regions effectively competing to amplify activity and initiate a burst. The region that successfully triggers a burst and subsequently recovers from its activation has a high probability to initiate the next burst. However, other regions may have sufficiently recovered during this period, assuming the role of initiators and becoming the new leaders of network activation.

Within the noise focusing framework, spontaneous activity ranges from weak, unstructured fluctuations to episodes of strongly synchronized, network-wide bursting (Hernández-Navarro et al., 2021). Inter-burst activity consists of locally correlated fluctuations that may propagate over limited regions of the network but are insufficient to trigger a full burst. In contrast, network-wide bursts arise when these fluctuations are amplified and succeed in recruiting the entire neuronal population. In the context of our work, the local EC—constructed from pairwise causal influences among neurons—captures information of the overall bursts initiation map (Orlandi and Casademunt, 2017), but does not distinguish between inter-burst correlated activity and burst-initiating events. This lack of separability may explain the limited correlation between *P*_IZ_(*x*, *y*) and *P*_EC_(*x*, *y*) distributions. In addition, EC is computed exclusively from activity within the recorded area, while IZ also considers events originating outside the recording area, biasing the probability density. Moreover, long-range anatomical connectivity may also influence the emergence and localization of IZs; however, these contributions are not explicitly captured due to the projection of network interactions onto a two-dimensional spatial probability density.

It is important to note that IZs cannot be created de novo, since assemblies of neurons capable of triggering collective events require specific topological features—such as feedforward and feedback loops, and connectivity-inherited metric correlations (Orlandi et al., 2013)—that cannot be overtly reshaped or modified by electrical stimulation over the course of an experimental session. We therefore hypothesize that the delivered electrical stimulation may alter synaptic weights sufficiently to perturb the coupling between IZs and the rest of the network.

Interestingly, the competing dynamics described by the boxing model are not exclusive to in vitro cultures but also emerge in assemblies in the tectum, where it appears to provide an advantageous mechanism for visual detection in noisy environments (Romano et al., 2015).

### The importance of connectivity and neuronal excitability

Previous studies have investigated alterations in bursts’ IZs and the underlying network connectivity in response to an electrical stimulation. Bursts—or more broadly, avalanches of activity involving a varying number of participating neurons—can be viewed as spatiotemporal patterns of structured information flow (Orlandi et al., 2013). These patterns are typically stable over time, i.e., reproducible and difficult to suppress, and in principle are modifiable through external stimulation. Thus, bursts were therefore proposed to serve as a potential neuronal substrate for memory retention and information transfer within cortical networks (Madhavan et al., 2007). In this regard, we note that understanding the mechanisms underlying the onset and modulation of neuronal bursts is not only relevant for in vitro networks, but also provides valuable insights into the impact of perturbations in neuronal activity in vivo. For instance, Curic et al. (2024) observed that the location of IZs shifts depending on the type of anesthetic administered, indicating that the silencing of specific connectivity pathways affects large-scale avalanche patterning.

In this context, high-frequency stimulation was shown to reshape neuronal activity patterns and functional connectivity (Poli and Massobrio, 2018), whereas Jordan et al. (2024) reported subtle shifts in the regions where activity originated in organoids following electrical stimulation. On the other hand, Jia et al. (2022) found that IZs were correlated with specific network connectivity metrics, such as betweenness centrality. In our data, the spatial overlap between IZs and EC was limited, although statistically significant. For EC, we note that groups of neurons with strong local EC and high betweenness centrality may trigger isolated activity but not bursts. Indeed, IZs showed a stronger correlation with spectral centrality, whose PDF maxima are consistent with the presence of rare active phases driven by structural inhomogeneities (Moretti and Muñoz, 2013).

An additional ingredient playing an important role in IZs patterning is the excitation–inhibition (E/I) balance. Inhibitory neurons are crucial for synchronizing neural activity (Voigt et al., 2001) and stabilizing propagating firing patterns (Mongillo et al., 2018). Nevertheless, this does not mean that they are igniting collective activity. Indeed, it has been reported that IZs correlate with a high neuronal density and with a lower number of inhibitory neurons (Feinerman et al., 2007). Therefore, we speculate that manipulating the E/I balance, for example by blocking GABA_A_ inhibitory receptors, could substantially remodel the spatial organization of IZs and EC.

Changes in intrinsic neuronal excitability may also influence the emergence of population bursts. For instance, persistent sodium currents can increase neuronal excitability and enhance the probability that certain neurons trigger network-wide activity (van Drongelen et al., 2006; Zendrikov and Paraskevov, 2024). In this context, intrinsic properties may contribute to burst initiation, while synaptic connectivity shapes the subsequent propagation and spatiotemporal organization of activity across the network (Suresh et al., 2016).

### Leader neurons and IZs

An alternative view to the noise focusing phenomenon to understand the presence of IZs in neuronal cultures is the concept of “leader” or “pacemaker” neurons (Feinerman et al., 2007; Eckmann et al., 2008), which are understood as those highly active neurons that consistently initiate or precede network bursts. The hypothesis of highly active and resilient leaders was supported by evidence both in vitro (Kuebler et al., 2015) and in vivo (Kobayashi et al., 2024).

However, it remains unclear whether neurons with high FRs are indeed responsible for initiating bursts. For example, Zendrikov and Paraskevov (2024) suggested that burst nucleation may depend on non-pacemaker neurons characterized by high subcritical excitability and low activity during IBIs. Interestingly, these neurons are proposed to possess strong incoming and outgoing synaptic connections (Eckmann et al., 2010). In the context of our data, those neurons with a high number of effective connections could therefore be considered candidates for the “leader” role, which may explain the strong spatial correspondence observed between IZ and EC spatial patterns.

Local fluctuations in neuronal density and connectivity have been correlated with burst initiation in in vitro cultures. When such inhomogeneities exhibit a high degree of structural recurrence, strong synaptic connections, and sub-millisecond temporal lags—the resolution used in our study—they show a strong association with IZs (Lonardoni et al., 2017), although these zones do not necessarily coincide with regions of elevated firing rates (Okujeni and Egert, 2019), making it challenging to fully characterize leader neurons in relation to broader network properties.

An advantage of the noise focusing interpretation is that it does not solely rely on structural inhomogeneities within the network but also on the amplification and focalization of noise (Orlandi et al., 2013), although such structural inhomogeneities—e.g., topographical obstacles (Montalà-Flaquer et al., 2022)—may affect where this focusing occurs. Rather, noise focusing offers a mesoscopic perspective that emphasizes network-level dynamics over the fine details of individual neurons, making it more appropriate for describing the clearly non-local alterations induced by the electrical stimulation. This framework is indeed compatible with the boxing model: upon stimulation, only the synaptic weights—or even just noise levels—are modified, naturally reshaping the IZs without the need to destroy existing ones or generate new ones from scratch. A network can inherently support multiple IZs, and their modulation does not require structural rewiring but can instead be achieved through subtle adjustments to existing parameters, such as synaptic coupling.

### Activity patterns and preferred dynamical states

While our work focused on characterizing the displacement of IZs following stimulation, we did not analyze the spatiotemporal structure of the bursts themselves before and after the perturbation, as explored in other studies, e.g., in the context of network maturation in vitro or the presence of connectivity anisotropies (Montalà-Flaquer et al., 2022). Such an analysis could provide insights into the preferred spatiotemporal propagation patterns of activity within the cultures, and whether stimulation drives the network toward a new set of preferred dynamical states. For instance, Pasquale et al. (2017) explored how external stimulation does not override the network’s intrinsic organization; instead, it tends to evoke activity patterns that are consistent with the network’s endogenous (spontaneous) dynamics, particularly those originating from dominant IZs. This line of analysis is conceptually related to the notion of a “free energy landscape” (Beer and Barak, 2024), where the spatiotemporal activity of neuronal cultures is viewed as evolving within a rugged, degenerate energy landscape. In this framework, the applied electrical stimulation—or, more generally, any form of chemical, electrical or physical perturbation—can be interpreted as an energy injection that enables the system to escape from its current state. Once the stimulation ceases, the system relaxes into a new energy minimum that may not coincide with the original state. Due to the multitude of possible configurations, the landscape is highly degenerate, i.e., it contains many local minima. Through homeostatic mechanisms, the system may restore its overall energy level, yet settle into a different basin of attraction. In this scenario, the observed shifts in IZs location may reflect the transition toward other foci that were already latent within the pre-existing network landscape, potentially mediated by activity-dependent potentiation and de-potentiation of synaptic pathways.

In this context, the heuristic notion of free energy, loosely defined here, can be interpreted in terms of FRs or synaptic weight distributions. These measures did not show significant differences between the pre- and post-stimulation phases. However, the change in network state is evident through the displacement or emergence of new IZs, a phenomenon that does not necessarily entail a measurable free energy cost. Indeed, we observed that 24 h after stimulation, the cultures sometimes returned to a state resembling the pre-stimulation phase, suggesting that stimulation can transiently drive the system into a metastable state. Nevertheless, in other cases, the dominant “Post II” IZ was maintained, while in additional instances it shifted to a different region, indicating that no single consistent trajectory was observed across experiments (Fig. 4-2). Conversely, in cultures where stimulation failed to induce any observable changes, this may reflect a system trapped in a deep energy well, with barriers too high to overcome within the timescale and energy provided by the perturbation.

To conclude, in this work we investigated the displacement of IZs and changes in EC in HD-MEAs following an external electrical stimulation. While IZs emerge as markers of perturbations, they do not reveal the specific nature of these perturbations, which may arise from changes in connectivity, excitability, or intrinsic noise. Our results underscore the difficulty of guiding a neuronal network toward a desired dynamic state or of exerting precise, ad hoc control over the spatiotemporal patterns of neuronal bursts. Our experiments also raise important considerations regarding the challenges of training and maintaining neuronal cultures based solely on brief periods of electrical stimulation. This limitation suggests that more sustained stimulation, closed-loop feedback control (Shahaf and Marom, 2001; Le Feber et al., 2010; Kumar et al., 2016), or multimodal approaches may be necessary to achieve controllable network dynamics.

## Synthesis

Reviewing Editor: Adrien Peyrache, McGill University Decisions are customarily a result of the Reviewing Editor and the peer reviewers coming together and discussing their recommendations until a consensus is reached. When revisions are invited, a fact-based synthesis statement explaining their decision and outlining what is needed to prepare a revision will be listed below. The following reviewer(s) agreed to reveal their identity: NONE.

This study examines the extent to which electrical stimulation can induce rewiring at the neuronal level. To address this, cortical cells were cultured on a high‑density multielectrode array, allowing precise monitoring and manipulation of activity. The manuscript was reviewed by two experts, both of whom emphasized the importance of the question and the suitability of the in vitro approach, given the practical limitations of probing such mechanisms in vivo.

They noted that the work is carefully executed, clearly presented, and supported by rigorous analysis. Although the effects of stimulation on connectivity appear limited, the reviewers stressed that reporting such outcomes is valuable in a field where previous findings have been inconsistent. At the same time, they pointed out limitations that affect the strength of the conclusions, for example, the inability to precisely control the new initiation zone of network bursts despite being able to shift it. Both reviewers view the study as a meaningful contribution to ongoing efforts to understand and control network dynamics, while recommending clarifications and refinements in a revised version.

Major concerns:

(1) Publications from several laboratories report a significant effect of the anatomical network structure on the efficacy and propagation of stimulation (e.g. from Marom, Soriano, Egert labs), such as by designed or spontaneous clustering and segmentation of the network. This is further supported by computational models such as from the Lizier lab. The current manuscript, however, does not report anything about the networks' structure and implicitly treats them to be structurally homogeneous in many procedures and interpretations, which they most likely are not.

(2) The impact of stimulation has been shown to depend on the functional state of the network at the time of stimulation (phase-dependency). Stimuli may thus vary highly in impact depending on when they are applied relative to the current activity (see reports from LeFeber, Egert and Marom labs, for example). This is not taken into account in the experiments and discussion.

(3) While by far most of the network is on-chip, unless the network size has been limited specifically, the boundary of the network may be outside of the recording area. This boundary typically has pronounced recurrent connectivity, which would affect burst initiation and propagation, and the region is not accessible to direct stimulation. There is no mention of this factor and how this affected the identification of IZs in the manuscript.

(4) In the introduction (lines 75-77), any effects of stimulation on IZ shift are explained as "This reorganization involves the potentiation and depotentiation of synaptic pathways, leading to the emergence of new dominant foci that were already latent within the pre-existing network architecture." The manuscript presents no evidence for this whatsoever. Without further support by data this is a conjecture, however plausible it may be, that should at best be presented in the discussion and as a conjunctive.

(5) While stimulus location is well defined by the electrode coordinates, the neurons stimulated are not. Any axon or dendrite passing the stimulation electrode could be activate ortho- or antidromically, with their somata sitting further away. Via collaterals, induced spikes may propagate to synapses at any location. In contrast the largest spikes will be picked up by electrodes close to the soma or axon hillocks. The population recorded in some area is thus not necessary the one stimulated. This should be considered in the discussion.

(6) The analysis focuses on the location and number of IZ. Yet, the succession of their activation might also be affected by stimulation, might be particularly sensitive because of induces synaptic depression and may indicate long-term vs. short-term changes of the effective connectivity. A complementary perspective on the organization of burst initiation is provided by the boxing model (Feinerman et al., 2007), which captures the competition between multiple IZs, predicting two dynamical regimes: one where a single zone dominates over its competitors, and another where several IZs coexist. Stimulation my then change the interaction but not the location of IZs.

(7) It is mentioned several times that there is no statistical difference between 'Pre' and 'Post I'. However, only the differences between 'Pre' and Post II' are shown. Corresponding measures (for IZ, EC, IBI) for 'Pre' and 'Post I' should be added. This can potentially be in the extended data.

(8) It would be useful to show more raw data to get a better feeling on how network dynamics is affected by external stimulation. For example, the distribution of a) the firing rates of all neurons (and not only the variation of FR as in figure 7), b) the interneuronal correlation, and c) the variability (e.g. Fano Factor), within and outside network burst, and at the different epochs of the experiment ('Pre', 'Post I' and 'Post II').

(9) The table in the extended data should include the location of the IZ. A schematic would be useful to read the coordinates.

(10) A detailed legend should be provided for the extended data table. It is not clear what 'dissection' means. Does it mean that the 5 lines with 'dissection=1' are from the same batch of neurons that have been plated together? Also, when several DIV are indicated, does it mean that the same stimulation electrodes were used at each experiment?

The number of stimulation electrodes and their distance to the IZ should be provided.

(11) The discussion states that 'A key feature of our experimental setup is the ability to freely control both the number and location of stimulated electrodes'. However, it is unclear whether these 2 parameters were varied in a systematic manner. Then, it is said that 'two main configurations' were tested. However, there is no results that showed a similarity or a difference between these two configurations except that 'we observed no differences...'. Given the amount of statistical analysis that were carried out to characterize differences (or lack of) between IZ or EC, it is somehow disappointing that there was no clear analysis to compare the different stimulation paradigms. Also in regard to the previous point, the table is useful, but the reader has no idea whether the actual experiment corresponds to one or the other of the stimulation configuration.

To sum up, a more systematic analysis of modulation of network dynamics should be provided regarding the stimulation site (i.e. separate the data into 2 groups: stimulation within and outside IZ).

(12) The discussion states that 'we observed that 24 hours after stimulation, the cultures often returned to a state resembling the pre-stimulation phase'. However, there does not seem to be data supporting this statement. This should be supported by further analysis.

Minor concerns:

The term 'avalanches' is used quite loosely in the manuscript but is elsewhere more often defined as in the context of avalanche statistics as used by Beggs and Plenz. This should be clearly defined what it is meant by the term in this manuscript to avoid confusion. Since it seems to be used to indicate network bursts, it would be better to stick with the latter term throughout.

Please provide an estimate of the spatial extent of the electrical stimulation and relate it to the distance of the stimulation site to the IZ.

It seems at some point (especially in the discussion) that conclusions are drawn from a few exemplary data. It might be worth to be more general in the conclusion.

In figure legends and elsewhere, please mention the 'n' when providing p-values.

Fig. 2: How much of the network is outside of the recording area? Panel (C): Explain why some electrodes are marked in darker red (badly visible though) in the caption or a suitable legend.

Fig 3B: Explain the red markers in the bottom-right panel.

Fig 6a: Was there any cell damage by stimulation? Caption (A) implies a causality that changes were stimulation-induced. There is no record of how often such changes occur spontaneously I the course of hours. Please add color bars and linear of log color scales for quantitative comparisons.

L122: "Action potential waveforms" typically refer to transmembrane potentials, which were not recorded. Please use a different term or clearly define what these "waveforms" should look like.

Same line: What does "participation in global network bursts" mean. Global activity is calculated in section 4.6.2 but there is no exclusion criterion. As described there, activity on a single electrode might, in principle, be used to calculate global activity.

Please define exclusion/inclusion criteria.

Section 4.6.3, 1st paragraph: The procedure to identify IZ is somewhat obscure.

- What were the criteria when "visually inspecting" a random subset?

- Why half of the detected bursts and at random? Random selection can lead to spurious biases. Where these controlled for? Using only a subset probably prevents the analysis of IZ sequences.

- Why were only 'wave-like propagation patterns' included? This implies a specific connectivity structure (mostly short-range and homogeneous) that thus is preselected. Such IZs could be particularly stable against stimulation. Besides, if those events were preselected, the sampling of bursts was not random.

The procedure in described in this paragraph seems rather heuristic for manageability but is not controlled for statistical impact (at least not reported).

Section 4.6.4: This approach assumes a homogeneous composition of the sample at each electrode w.r.t. the number of neurons participating and a temporally homogeneous and consistent composition. This would be violated if neuron density around some electrode and neuron properties varied. Some electrodes may then pick up spikes from many, others from just one neuron - which would affect burst participation probability and hence IZ localization as well as EC calculation.

- Was spike sorting performed (which could be done well with these arrays)?

- If not, how did the authors ensure homogeneity of the sampled data?

L207: Why is a distance-dependent weight used? This again presumes homogeneity and predominantly local connectivity.

L265 typo "... in a window ..."

L276 and elsewhere: The description here does not take into account the succession of IZ activation. For example synaptic depression by stimulation could change the sequence of IZs dynamically, without changing their location over longer periods. The fraction of bursts initiated at a particular IZ may change in the same way.

L295: How would this be affected by changes in variability of IZ activation?

L309-319 vs 320-321: These paragraphs seem inconsistent. If (L309) there is a discrepancy between IZ and EC changes, why would (L320) EC and IZ align?

If homeostatic responses smooth out reconfiguration, why would EC and IZ correlate well?

L334 onward: This section should also take into account what Okujeni et al reported in their Frontiers paper, where regions with above or below average activity were less likely to be IZs. They discussed synaptic short-term depression as possible factors for burst initiation, or rather suppression. This be affected by convergence and recurrent connectivity on a local set of neurons. Areas with high activity may indicate such conditions and be more strongly suppressed after bursts.

L338 and elsewhere: avalanches not defined and misleading because of possible confusion with avalanche in Plenz context

L344 Please explain why this quantitative comparison is permissible.

L362-363: This sentence doesn't make sense as it is.

L370: the spatial resolution after all the Gaussian smoothing would anyway be too low to show anything else. What would be alternatives that could be detected with a more fine grained analysis?

L377: sentence structure

L386: It could be that the network re-balances by the time of Post II. To make such a statement would require to analyze Post I vs. Post II at least.

L397: This refers to average (smoothed) effects. Please clarify.

L404: add culture ID

L413/414: That high rates do not indicate IZ was reported in Okujeni et al already. You might want to use this as supporting literature.

L457: While mesoscale inhomogeneities are probably relevant, their effect on the dynamic state of such networks might be more important, or rather the important consequence of the inhomogeneities. For example, the propensity for super-/subcritical states could change, which would affect network recruitment. Compare, for example, Okujeni &Egert 2023. On the other hand the degree of synchronization and absolute peak firing rates during an event may affect synaptic depression (degree and spatial distribution, see also comment on L334).

L505: Which data in the ms prove "non-local" mechanisms specifically?

L513: Okujeni '19 does not require strong connectivity for BIZ as would be recovered from EC analysis!

L520 and surround: Noise focusing presented as if in a homogeneous network (though that is not a necessity). Please discuss the contribution of the anatomical network structure.

L533: typo: "stimulation"

L532-535: Fig 3B is just one example, which is not sufficient to make such a claim.

L542: Why "coordinated", what does this mean?

L563-574: Could you make this section more specific and focused?

L594: This relation is not symmetric: While not necessary, structural inhomogeneities could nonetheless contribute to noise focusing as well as IZs.

L636: Please reference work by LeFeber et al and Kumar et al on closed-loop stimulation.
